# Central Dental Association of Northern New Jersey

**Published:** 1884-09

**Authors:** B. F. Luckey

**Affiliations:** Sec’y.


### CENTRAL DENTAL ASSOCIATION OF NORTHERN NEW JERSEY.

A regular meeting of the Central Dental Association of Northern New Jersey was held at the house of Dr. C. F. W. Bödecker, of New York, at 60 East Fifty-eighth Street, on Monday, April 20, 1884, the President, Dr. Barlow, of Jersey City, in the chair.

Upon motion of Dr. Levy the regular order of business was suspended, and Prof. Abbott proceeded to address the meeting.

(Dr. Abbott's address will be found on page 475.)

### DISCUSSION.

*Dr. Watkins*—I feel that I am well paid for attending this meeting. I would, however, like to have Dr. Abbott tell us how he caps exposed pulps, and what his treatment is when he amputates a part of the tissue.

*Dr. Abbott*—I treat the case as simply as possible, and I use as little in the way of medicines as will accomplish the result. If I find the pulp of the tooth exposed, that is, a recent exposure, I at once cap it. I do it in the following manner: When I have prepared the cavity and everything is ready, I place a little bit of cotton saturated in creosote directly upon the exposed point, and another larger piece over that, producing a slight pressure upon the pulp. This I allow to remain until my capping material is prepared. I then remove the cotton and place the cap in position. I use my cement quite thin, and allow it to gently flow over the exposed point of the pulp. Then, if I am in a hurry, and wish to fill with a substance that requires pressure, I place a small piece of platinum over the cap, and proceed with my work. The cement I am now using for this purpose is prepared by the Union Tooth Company.

If the patient comes back with any disturbance (which, however, does not occur in more than one case in twenty), I paint the gum with aconite and iodine ; this I do two or three times. If that does not relieve it, I remove the filling and apply creosote again, upon a little cotton, and cover that with gutta percha and wax for twenty-four hours. If there is no pain I go on and cap again, the same as before. If any of the pulp has died I take a sharp bur drill, large enough for lateral cutting, so as to prevent the sudden injury to the pulp, and cut until the patient feels a like pain. I then wash out all the dead matter and bits of tooth with warm water and salt, and apply creosote upon cotton, and cover over with gutta percha and wax for a few hours, or perhaps two or three days, and if everything is quiet, I go on and cap as before.

When a small portion of the pulp only is alive in a pulp canal, I carry a little twist of cotton saturated with creosote against that, leaving the end of the twist sticking out of the canal into the pulp chamber, put a little cement in the bottom of the cavity, and fill. The twist of cotton is left in this position, so that should occasion require it can readily be removed.

*Dr. Watkins*—Would it not be the fact that if liquid gutta percha were used as a root filling it would flow around part of the pulp and remain under the root and seal that part up; and if so, is there danger of that part becoming putrefied?

*Dr. Abbott*—I think not.

*Dr. Palmer*—I have heard this question of filling the roots with oxy-phosphate and oxy-chloride of zinc discussed, and it is said that they would be liable to deteriorate. I want to know what is the experience of those present in regard to this.

*Dr. Abbott*—I don't believe any such thing. I have now in my possession a molar tooth through which three drill-holes were made, and cotton saturated with oxy-chloride of zinc penetrated the alveolus from one-eighth to a quarter of an inch in depth, where it remained a year or more, and no deterioration took place.

The meeting, upon motion, was adjourned.

CONTINUATION OF DISCUSSION AT THE MAY MEETING—INCIDENTS OF OFFICE PRACTICE.

*Dr. Watkins*—I have a case in which I am very much interested. I would like to describe it here. A young lady came to my office from New York with the intention of having two teeth bleached and returning the same day. They were both dead—one beginning to ulcerate.

They were the upper and lower left central incisors. There seemed no particular cause for the death of the pulps. The upper one had a very small approximal filling. The lower one had neither filling nor cavity. I drilled into each of them and removed the putrefied pulps. The lower one gave no pain whatever. I disinfected it thoroughly by carrying iodoform through the canal to the apex, and filled the end of the canal with gutta percha, then bleached the tooth with Labaraque's solution and alum, and filled nearly full with oxy-chloride of zinc and covered it with gold, painting the gum with tincture of aconite and iodine. I then disinfected the upper one, but the pain in that did not cease; painted the gum, and advised her to remain over night; continued the painting every two or three hours, which would give relief for a few minutes each time. During the night the lower one began to cause pain. In the morning it got its full share of aconite and iodine, but apparently without benefit. Then the capsicum bags were tried, which gave relief for a short time. I was obliged to go to New York that evening, and accompanied her to the train. Almost immediately the pain returned, and I think I never saw such agony, and all of this after being thoroughly dosed with iodoform, aconite and iodine, and capsicum bags. Upon arriving in New York we called at Dr. Lee's office, and after consulting together and trying different remedies without relief, I decided to try the remedy recommended by our old friend, Dr. Atkinson, which is to insert a sharp knife through the gum, as near the end of the root as possible; with a firm, steady hand press it into the cementum, and draw down or up, as the case may require, almost to the edge of the gum. I applied crystallized carbolic acid to the lower gum first, just where I wanted to cut it, which destroyed nearly all sensitiveness, and hurt her very little. Just a slight flow of pus, with considerable congested blood, passed out in a moment. As soon as she had removed the blood from her mouth she begged me to cut the other one, exclaiming that it felt good, which I did in the same manner, and the pain disappeared entirely. Five days have elapsed, and to-day she wrote me that she had suffered no pain from the teeth since.

*Dr. Cosad*—What followed the lancing?

*Dr. Watkins*—In the lower one just a little pus and considerable congested blood; in the upper one nothing but blood.

*Dr. Luckey*—I have a case that will illustrate the forbearance of nature toward *some* foreign substances, and also what can be done with liquid gutta percha as a root filling. Last Friday morning a patient came to me with a tooth that I had been treating for abscess. It was a superior central incisor, and there was a large, fistulous opening in the gum. There was no sign of inflammation, so I determined to fill the root at that time. I applied the rubber dam, washed out my medicines, dried everything nicely, and then filled it carefully with gutta percha dissolved in chloroform. On removing the rubber dam, and raising the lip I was surprised to find my beautiful gutta percha sticking out of the fistulous opening. I asked the patient if she felt any pain. She said she did not—even felt comfortable. I took a spoon excavator and went through the opening clear to the root, and removed every particle of the gutta percha, painted the gum with aconite and iodine, and dismissed her until this morning. When she returned she said the tooth had been very comfortable, and had given her no trouble whatever. The first thing I did was to raise her lip and examine the fistula, and there, to my astonishment, I found three or four good-sized globules of gutta percha along the track, which must have come from the root since her last visit. I removed them and then filled the tooth permanently with gold. I think that is pretty good evidence that nature will tolerate gutta percha so long as there is room for it, and it does not cause undue pressure on the surrounding parts. B. F. LUCKEY, D. D. S., *Sec'y.*

